# Acute Brachial Artery Occlusion on Point-of-Care Ultrasound in the Emergency Department: A Case Report

**DOI:** 10.5811/cpcem.2022.9.57482

**Published:** 2022-11-05

**Authors:** Anisa Y. Mughal, Patrick Kishi

**Affiliations:** * Creighton University School of Medicine, Department of Emergency Medicine, Phoenix, Arizona; † Mayo Clinic, Department of Emergency Medicine, Phoenix, Arizona

**Keywords:** case report, brachial artery occlusion, point-of-care ultrasound, vascular ultrasound

## Abstract

**Introduction:**

This is a case report of an acute right brachial artery occlusion found on point-of-care ultrasound in the emergency department (ED) that illustrates the developing role of ultrasound in rapid differentiation and identification of acute vascular emergencies.

**Case Report:**

An 87-year-old male with a past medical history of coronary artery bypass graft presented to the ED with acute right upper extremity pain, with point-of-care ultrasound (POCUS) findings consistent with acute right brachial artery occlusion.

**Conclusion:**

Arterial occlusions are vascular emergencies that can be rapidly identified on POCUS.

## INTRODUCTION

Acute arterial occlusion is a medical emergency caused by a disruption in arterial flow. The occlusion may occur from thrombosis or embolus. Clinical presentation and symptoms vary based on the artery affected. These occlusions ultimately are responsible for a significant incidence of disability and death (eg, myocardial infarction, stroke).

Acute limb ischemia affects approximately 10–14 patients per 100,000 annually and more commonly occurs in the lower extremities.^1^,^2^ The brachial artery is located in the upper extremities and is commonly used to measure blood pressure. Acute embolization of the brachial artery is most commonly cardiac in origin.^2^ Trauma, aneurysms, and iatrogenic injuries following cardiac catheterization may also cause acute thrombosis.^3^,^4^ Several studies have identified upper extremity arterial occlusions after coronary catheterization; however, there are few reports of unprovoked brachial artery occlusion.^1^,^5^,^6^

When considering brachial artery occlusion in patients, the diagnostic workup should include a thorough physical exam, point-of-care ultrasound (POCUS), and coagulation laboratory studies.^7^ A computed tomography (CT) angiogram can be considered if POCUS and clinical exam are insufficient to provide this time-sensitive diagnosis.^7^ In this case report we describe an acute brachial arterial occlusion in an elderly male presenting with right upper extremity pain.

## CASE REPORT

A left-handed, 87-year-old male unvaccinated for COVID-19 with a past medical history significant for a four-vessel coronary artery bypass graft in 2017, not on any anti-coagulation medications, and ischemic cardiomyopathy presented to our emergency department with acute-onset right upper forearm and right-hand pain. The patient reported severe, excruciating pain in his right upper extremity starting at the level of the elbow extending to his distal fingertips upon waking approximately two hours prior to presentation. He also reported paresthesias that were most prominent in his right hand. Prior to arrival, the patient took two full-strength aspirin tablets (325 milligrams), which did not provide any relief. The review of systems was otherwise unremarkable.

The patient’s initial vital signs were notable for temperature 36.6° Celsius, heart rate 89 beats per minute, respiratory rate 16 breaths per minute, blood pressure 189/82 millimeters of mercury, and oxygen saturation of 100% on room air. On physical exam, he appeared uncomfortable but was not toxic-appearing or diaphoretic. There was no palpable pulse in the right brachial artery, the right radial artery, or in the right ulnar artery. Examination of the right upper extremity demonstrated a cold and pale limb from the level of the elbow to the hand. All compartments were soft. Motor strength and sensation were intact.

Due to the concerning physical exam findings, a POCUS was performed by the emergency physician. The ultrasound findings demonstrated an arterial occlusion in the right brachial artery, with visible clot burden (Image, Video 1). Color Doppler revealed no evidence of blood flow from the point of the occlusion in the antecubital fossa and distally (Video 2). Vascular surgery was immediately consulted, and a heparin drip was ordered, along with analgesic medications and intravenous fluids. While laboratory studies were pending, vascular surgery evaluated the patient, and a decision was made to take the patient to the operating room for emergent repair with a right brachial artery thrombectomy. He also tested positive for severe acute respiratory syndrome coronavirus 2 (SARS-CoV-2) but did not report any related symptoms.

Ultrasound was used intraoperatively to identify the proximal extent of the thrombus within the brachial artery. The brachial artery appeared clear of thrombus and embolus approximately three finger breadths proximal to the antecubital fossa. The clot was removed, and at the conclusion of the procedure the patient had palpable radial and ulnar pulses. Physical exam indicated the patient had Doppler signals in the right palmar arch after thrombectomy. Postoperatively, he reported that his pain and paresthesias had resolved.

The patient underwent further workup during hospitalization, which included a transthoracic echocardiogram demonstrating an apical left ventricular thrombus that was pedunculated and mobile. Ejection fraction was calculated to be 29%. A CT angiogram of the chest demonstrated no evidence of an ascending aortic or great vessel thrombus to explain embolization. The patient was continued on a heparin drip and subsequently transitioned to oral warfarin with a goal international normalized ratio (INR) of 2.0–3.0. He was discharged on post-operative day two with a 10-day course of therapeutic enoxaparin as a bridge until he reached a therapeutic INR. Chart review demonstrated no evidence of complications after discharge.

**CPC-EM Capsule**

What do we already know about this clinical entity?

*Upper extremity arterial occlusions occur less commonly than lower extremity arterial occlusions and are often traumatic or iatrogenic in origin*.

What makes this presentation of disease reportable?

*We describe an unprovoked brachial artery occlusion in an elderly male patient with asymptomatic coronavirus disease*.

What is the major learning point?

*Point-of-care ultrasound can quickly identify and diagnose acute arterial occlusions in the upper extremity*.

How might this improve emergency medicine practice?

*Point-of-care ultrasound can lead to rapid diagnosis of vascular occlusions and facilitates rapid treatment to prevent irreversible ischemic pathology*.

## DISCUSSION

Our report highlights a unique presentation of acute brachial artery occlusion in which POCUS led to a rapid identification and expedited treatment of this critical, acute vascular emergency. Clinical signs of arterial occlusion follow the common mnemonic of the six Ps: pulselessness, pain, pallor, poikilothermia, paresthesia, and paralysis.^7^ Sonographic signs of arterial occlusion include visible clot burden or a non-compressible vessel, and absence of color Doppler flow of the affected vessel.

The prevalence of acute arterial occlusion varies by anatomical location, risk factors, and gender.^2^ Upper arterial occlusion occurs less frequently than lower extremity arterial occlusion.^2^ Modifiable risk factors include smoking, obesity, high cholesterol, diabetes, and hypertension.^7^ Increasing age correlates with a higher incidence of arterial occlusion.^8^ COVID-19 may be a new risk factor in the development of arterial occlusion. In 2021, a nine-patient retrospective analysis was performed, demonstrating that patients with underlying conditions presenting with elevated inflammatory markers or D-dimers in the setting of SARS-CoV-2 infection were at a higher risk of developing acute arterial occlusion.^9^

Most cases of radial artery occlusion are studied in the context of a transradial catheterization approach and document strategies to reduce iatrogenic or post-procedural radial arterial occlusion.^6^ In this case, the extremity clot was likely a result of embolization from the ventricular thrombus demonstrated on formal echocardiography. A ventricular thrombus can form in vivo, or secondary to recent myocardial infarction, and is often the source of peripheral arterial occlusions.^10^ In patients presenting to the ED with arterial occlusion, it may be beneficial to perform a POCUS echocardiogram to evaluate for a thrombotic source.

## CONCLUSION

Point-of-care ultrasound can lead to rapid diagnosis of vascular occlusions. This is particularly important in identifying peripheral arterial occlusions that otherwise can lead to irreversible ischemic pathology. It is important to include acute thrombosis in the differential diagnoses in patients with recent or active SARS-CoV-2 infections, as it may lead to a hypercoagulable state.

## Supplementary Information

Video 1 Point-of-care ultrasound of the right brachial artery with an occlusive thrombus.

Video 2 Point-of-care ultrasound demonstrating absence of color Doppler flow in the brachial artery, indicating an occlusive thrombus.

## Figures and Tables

**Image:**
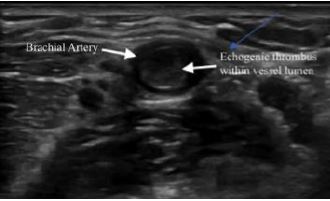
Point-of-care ultrasound image of proximal radial artery with an occlusive, non-compressible thrombus.
